# P-1393. Tuberculosis Awareness and Misconceptions in Asian American and Pacific Islander Communities

**DOI:** 10.1093/ofid/ofaf695.1580

**Published:** 2026-01-11

**Authors:** Kevin M Zhang, Noah Razak, Eloy Alibin, Nicole Delos Santos, Edward Seto, Xuan Man, Mai Vi Hoang, David Zhang, Raj Palraj

**Affiliations:** University of Washington, Seattle, WA; Issaquah High School, Issaquah, Washington; Community Health Care, Tacoma, Washington; Community Health Care, Tacoma, Washington; Community Health Care, Tacoma, Washington; Tacoma-Pierce County Health Department, Tacoma, Washington; St. Rose San Martin Dignity Health Medial Center, Las Vegas, Nevada; Virginia Mason Franciscan Health, Tacoma, Washington; Division of Public Health, Infectious Diseases, and Occupational Medicine, Department of Medicine, Mayo Clinic, Rochester, Minnesota

## Abstract

**Background:**

Stigma associated with tuberculosis (TB) and TB diagnosis is a major social factor in delay of diagnosis and non-adherence to TB treatment among Asian American and Pacific Islander (AAPI) populations. Main sources of stigma include misconceptions regarding TB transmission and lack of knowledge about symptoms or treatment.

**Figure 1.**
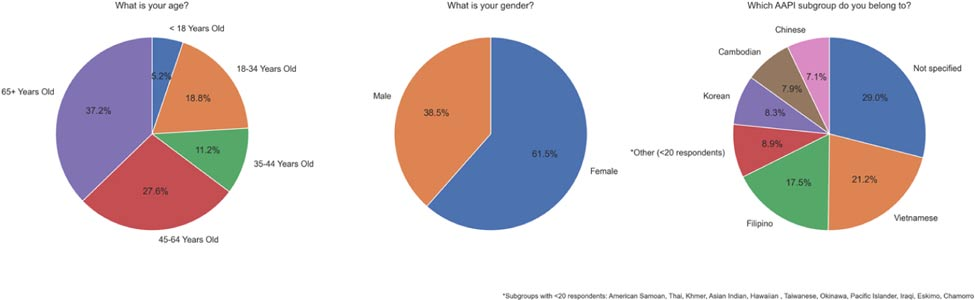
Respondent demographics Breakdown of respondent demographics by age, gender, and AAPI subgroup.

**Figure 2.**
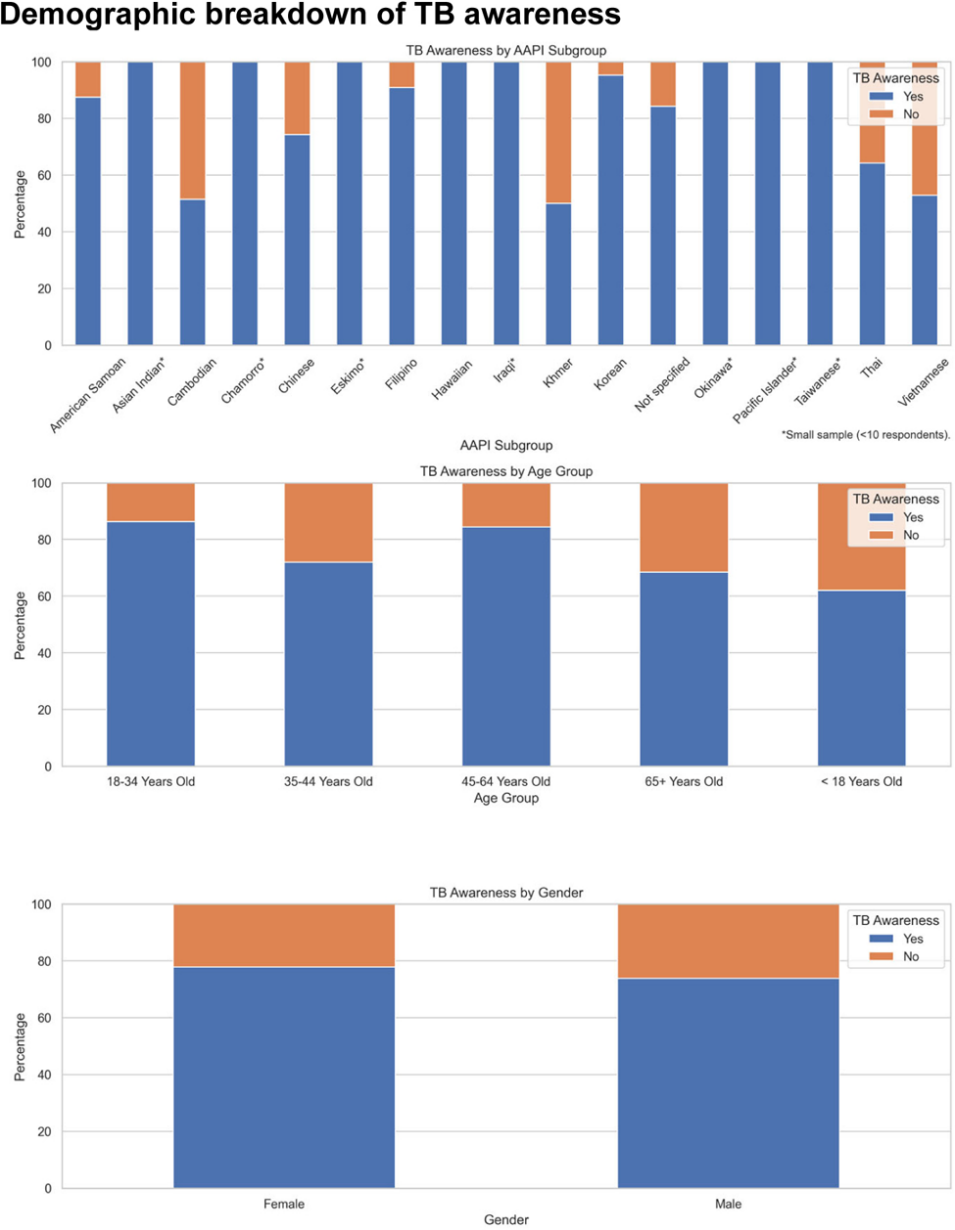
Demographic breakdown of TB awareness TB awareness by AAPI subgroup, age, and gender.

**Methods:**

A cross-sectional survey of 666 AAPI individuals analyzed TB symptom recognition, transmission misconceptions, and awareness in two AAPI community centers. Data were segmented by AAPI subgroup, metropolitan area (Seattle/Tacoma vs. Las Vegas), age, and gender.

**Figure 3.**
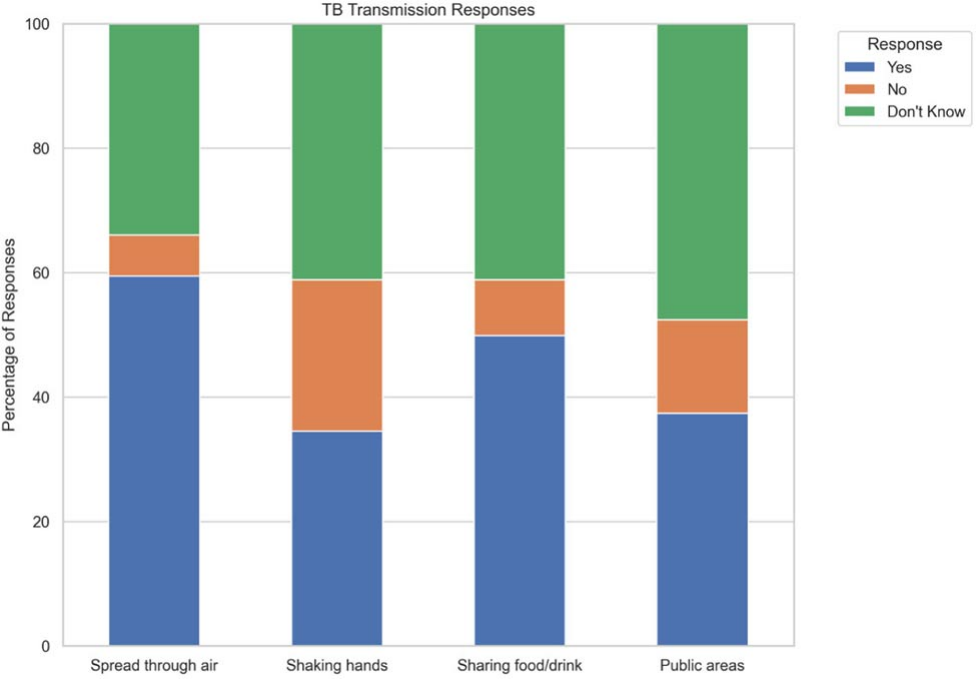
TB transmission beliefs.

**Figure 4.**
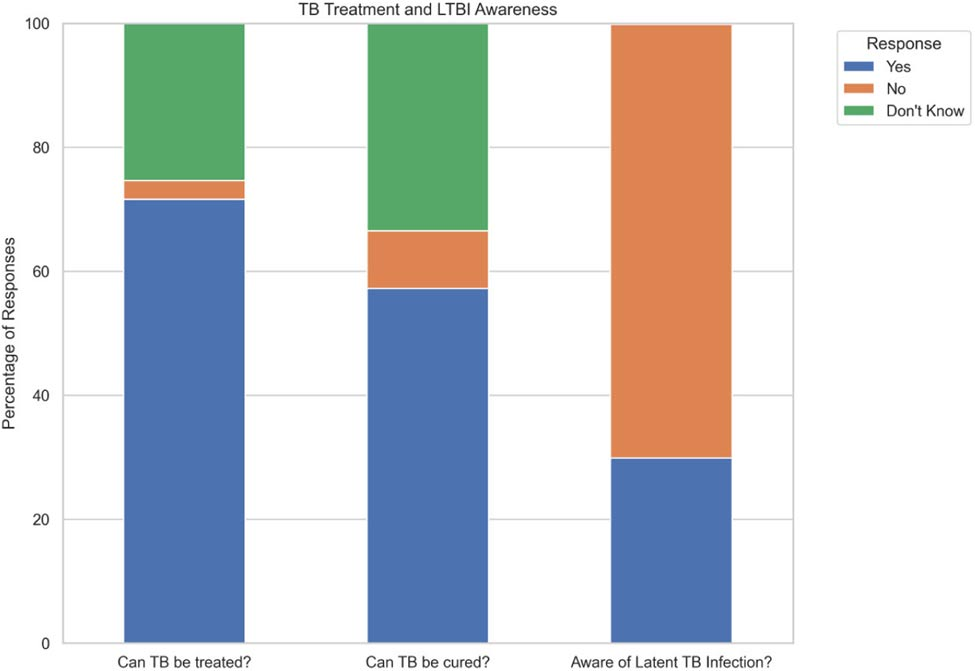
TB treatment, curability, and latent TB infection awareness.

**Results:**

Overall, 76.6% of respondents reported TB awareness, varying by AAPI subgroup. Awareness was highest among Korean (95%) and Filipino (91%) participants, and lowest among Vietnamese (52%) and Cambodian (50%) respondents. Regionally, TB awareness was similar between Tacoma (76%) and Las Vegas (79%).

Age and gender significantly impacted awareness. Respondents under 18 had the lowest TB awareness (59%), while those aged 18-34 had the highest (84%). Females reported higher TB awareness (79%) and symptom recognition (61%) than males (74% and 46%, respectively).

Symptom knowledge varied. 55% were aware of TB symptoms, but only 16% were able to recognize five common TB symptoms. Misconceptions about transmission also persisted broadly. Out of the 71% of respondents who were aware that TB is transmissible, many incorrectly believed TB spread via shaking hands (35%), sharing food/drinks (50%), or touching items in public areas (37%). The 45-64 and 65+ age groups were most prone to misconceptions about transmission.

While 72% of respondents were aware that TB could be treated, only 57% knew TB could be cured. 30% of respondents were aware of latent TB infection.

**Conclusion:**

Significant disparities in TB awareness exist in AAPI communities. Females demonstrated greater TB awareness perhaps due to cultural differences (“caretaker role”), while TB transmission misconceptions were prevalent among older individuals. Limited knowledge on LTBI and TB treatment represent significant barriers to effective TB control. Tailored educational efforts will be required to improve TB awareness and reduce misconceptions within AAPI communities.

**Disclosures:**

All Authors: No reported disclosures

